# A Deep Learning-Based Framework Oriented to Pathological Gait Recognition with Inertial Sensors

**DOI:** 10.3390/s25010260

**Published:** 2025-01-05

**Authors:** Lucia Palazzo, Vladimiro Suglia, Sabrina Grieco, Domenico Buongiorno, Antonio Brunetti, Leonarda Carnimeo, Federica Amitrano, Armando Coccia, Gaetano Pagano, Giovanni D’Addio, Vitoantonio Bevilacqua

**Affiliations:** 1 Bioengineering Unit of Bari, Istituti Clinici Scientifici Maugeri IRCCS, Via Generale Bellomo, 73/75, 70124 Bari, Italy; lucia.palazzo@icsmaugeri.it (L.P.); gianni.daddio@icsmaugeri.it (G.D.); 2 Department of Electrical and Information Engineering (DEI), Polytechnic University of Bari, Via E. Orabona, 4, 70125 Bari, Italy; vladimiro.suglia@poliba.it (V.S.); s.grieco1@studenti.poliba.it (S.G.); domenico.buongiorno@poliba.it (D.B.); antonio.brunetti@poliba.it (A.B.); leonarda.carnimeo@poliba.it (L.C.); vitoantonio.bevilacqua@poliba.it (V.B.); 3 Bioengineering Unit of Telese Terme, Istituti Clinici Scientifici Maugeri IRCCS, Via Bagni Vecchi, 1, 82037 Telese Terme, Italy; federica.amitrano@icsmaugeri.it (F.A.); armando.coccia@icsmaugeri.it (A.C.)

**Keywords:** gait recognition, gait disorders, inertial measurement units, deep learning, convolutional neural network, rehabilitation, bioengineering

## Abstract

Abnormal locomotor patterns may occur in case of either motor damages or neurological conditions, thus potentially jeopardizing an individual’s safety. Pathological gait recognition (PGR) is a research field that aims to discriminate among different walking patterns. A PGR-oriented system may benefit from the simulation of gait disorders by healthy subjects, since the acquisition of actual pathological gaits would require either a higher experimental time or a larger sample size. Only a few works have exploited abnormal walking patterns, emulated by unimpaired individuals, to perform PGR with Deep Learning-based models. In this article, the authors present a workflow based on convolutional neural networks to recognize normal and pathological locomotor behaviors by means of inertial data related to nineteen healthy subjects. Although this is a preliminary feasibility study, its promising performance in terms of accuracy and computational time pave the way for a more realistic validation on actual pathological data. In light of this, classification outcomes could support clinicians in the early detection of gait disorders and the tracking of rehabilitation advances in real time.

## 1. Introduction

Human locomotion is a symmetric motor action [1] that requires the involvement of the central and peripheral nervous systems actuating mechanisms to control limb movements, posture, and muscle tone. When this neuromotor process is compromised by either physiological decline due to aging or such pathological conditions as Parkinson’s disease (PD), abnormal walking patterns may be exhibited [2]. Therefore, monitoring locomotor behaviors is needed to evaluate whether and how an individuals’ motor state deviates from a healthy gait [3], and such difference may be an indicator of a gait disorder [4,5].

In the context of healthcare, the employment of systems for monitoring and recognizing gait allows one to record motor execution, identify a possible gait disorder, and provide feedback for the patients’ assistance [6,7,8,9,10]. In light of this, walking pattern recognition even has the potential to support clinicians for both rehabilitative and remote-monitoring purposes [11], aiming at the treatment of neuromotor pathologies, such as PD [12,13,14,15,16], cerebellar ataxia [17], stroke [18,19], and cerebral palsy [20].

Gait recognition (GR) is a human activity recognition problem that is directed to differentiate locomotion from other motor actions [3], whilst pathological gait recognition (PGR) may be regarded as a subfield of GR that is used to discriminate motor patterns between healthy and pathological ones in case of binary classification [4] or among different gait disorders [2,6,11,21,22,23,24]. Despite a binary classification being easier to accomplish due to the high deviation of pathological motor patterns with respect to normal gait [13,15,18], a PGR including different gait disorders allows one to discriminate even similar walking patterns [5], such as those of foot drop and hemiplegia.

The recognition of pathological gaits needs a large amount of data related to patients to be accurate, which implies that one needs to make the experimental subjects execute multiple repetitions of several motor actions [1,3]. However, this may be a highly demanding motor task in the case of actually impaired subjects due to their condition; for this reason, such individuals could perform the target motor pattern only a limited number of times or with longer breaks [9]. As a consequence, a dataset imbalance between healthy and pathological individuals would arise, thus introducing a bias in the classification; since this worsens the performance of automatic recognition, a higher number of patients needs to be involved [25]. Moreover, the specific disease severity influences the number of recruitable patients, which could be seriously restricted in the case of non-autonomous locomotion, and a constant supervision of clinicians is still required for the patients’ safety. Hence, the simulation of abnormal walking patterns by healthy individuals may provide a benefit to the performance of a PGR framework, considering their possibility to perform different trials of various walking actions. In so doing, the effectiveness of a classification pipeline can be evaluated prior to any investigations on actual pathological individuals [2]; this is similar to the concept of cross-subject domain adaptation [26], meaning that the model is pre-trained on abnormal walking patterns simulated by healthy controls before being finally tested on actual pathological data. Additionally, the PGR system could then carry out an early detection of abnormal walking patterns [5,27].

A pathological motor pattern may be replicated in different ways, such as fixing lower limb joints [4] or making the subject wear a shoe unilaterally [8]; however, such methodology could result in the individual’s discomfort during the execution, thus potentially leading to involuntary compensations that alter the simulation, and would require a complex setup to mimic various gait disorders. Therefore, experimental protocols addressing PGR are typically based on the simulation of such impaired motor behaviors without any physical constraint on the lower limbs [2,7,21].

A few studies trained artificial intelligence (AI) models, notably Deep Learning (DL) architectures, with inertial data regarding emulated gait disorder. Hence, a paucity in the literature has been found about the frameworks exploiting the simulation of abnormal walking patterns to train DL-based models for the final aim of recognizing actual pathological gaits. The objective of this work is to present the evaluation of CNN-based algorithms that aim to discriminate normal gait from abnormal human walking patterns, which are emulated by healthy subjects, by means of inertial data. Both the execution of normal gait and the simulation of multiple pathological behaviors are included in the experimental protocol. In addition, various sensor configurations (number and placement of IMUs) have been evaluated to estimate their impact on the model performance; their comparison has been analyzed to determine the proper tradeoff between the informativeness and the usability of the sensor placement for the sake of continuous usage oriented to clinical purposes entailing both remote monitoring and rehabilitation [3,7].

The remainder of this article is as follows. After reporting the state-of-the-art in Section 2, materials (i.e., the system and protocol for data collection) and methods (i.e., preprocessing operations, CNN-based architectures, and statistical analysis) are discussed in Section 3. Then, the results are shown and discussed in Section 4. Ultimately, in Section 5 conclusions about the conducted study are drawn and suggestions for future works are reported.

## 2. Related Works

Walking patterns for PGR can be recorded by means of such fixed sensors as optical motion capture systems [6], which provide experimenters with high-fidelity data on movement patterns during the gait cycle [28]. Among these devices, RGBD cameras have obtained comparable performance with respect to Vicon systems [6], which have a limited application due to their expensiveness [4]. RGBD cameras recognized activities of daily living (ADLs) with performances that were similar to or even higher than inertial wearable devices [29]. However, these devices are influenced by illumination and occlusions and are characterized by a limited coverage area [30]; in addition, their need for extensive preprocessing to prepare data for the actual classification pipeline [6] could be in contrast with real-time requirements of remote-monitoring applications [3]. Hence, such wearable sensors as inertial measurement units (IMUs) could be preferred for their portability, which allows for their usage in both outdoor and indoor environments [3,11].

Subsequently, motor behaviors can be recognized by Machine Learning (ML) models, which were trained on data related to either actual pathological individuals or locomotor disorders simulated by healthy subjects. For instance, Dolatabadi et al. [25] fed such ML models as a k-nearest neighbor (kNN) with the data coming from Kinect sensors to automatically discriminate the walking patterns of healthy subjects and individuals affected by either stroke or acquired brain injury. Ghobadi [7] trained a support vector machine (SVM) with IMU data to recognize a simulated foot drop behavior.

Notwithstanding, these pipelines needed a complex and time-demanding feature engineering stage prior to the actual classification [3,8]. On the other hand, Deep Learning (DL) architectures, such as convolutional neural networks (CNNs) [6,9,31], can be trained directly on raw data, thus avoiding manual feature extraction [3,21]. For instance, Oh et al. [9] exploited a 1D-CNN to recognize the activities of daily living performed by hemiparetic stroke patients and healthy controls, who wore IMUs on the wrist, forearm, upper arm, trunk, and ankle. However, such ADLs did not entail locomotor patterns, since they were conceived for investigating the asymmetry of upper limb motions of individuals after a stroke.

There exist a few studies that simulate pathological walking patterns to train PGR-oriented AI algorithms, whether ML or DL models, by acquiring inertial data. For instance, Robles et al. [2] utilized Artificial Neural Networks (ANNs) to classify simulated gait disorders (those of Parkinsonian, ataxic, and hemiplegic origins)by analyzing accelerations of the center of mass of healthy subjects. Ghobadi et al. [7] discriminated normal gait from mimicked foot drop (FD) by training a support vector machine (SVM) with the data coming from a single IMU placed on the subject’s right leg. Yin et al. [23] exploited two accelerometers to feed DL architectures, such as a CNN, with the aim of automatically classifying pathological gaits (e.g., hemiplegic, diplegic, and Parkinsonian gaits), which were emulated by healthy participants.

## 3. Materials and Methods

**Figure 1:**
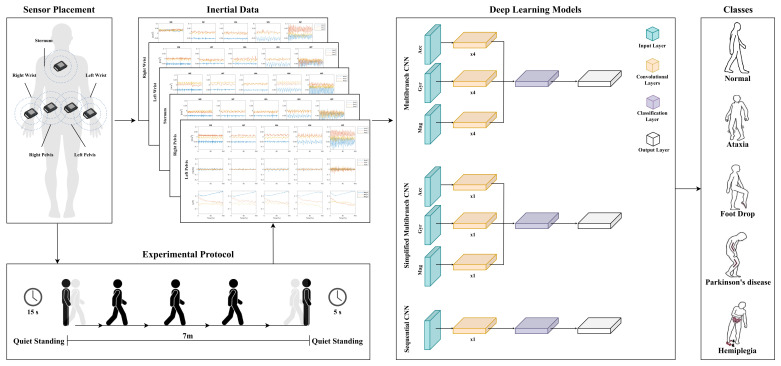
The presented framework is based on the acquisition of inertial data by means of five IMU sensors, whose components are given as input to each of three DL-based models, which return the label associated with the walking pattern.

The proposed framework for pathological gait recognition is reported in Figure 1. Herein, inertial data are first collected from five sensors placed on the human body during the execution of walking patterns, and then used to feed each of three DL-based classifiers, which differ in terms of architecture.

### 3.1. Data Collection

This section reports the individuals recruited in the experiments, the walking behaviors that were simulated, the IMU sensors used to acquire kinematic data, and the experimental protocol that was followed.

#### 3.1.1. Participants

Nineteen healthy subjects were recruited among the physiatrists and physiotherapists of IRCCS Maugeri (Bari, Italy) to guarantee a plausible simulation of pathological gaits. Proper balancing among males and females was guaranteed (i.e., 9 males and 10 females) to prevent the model from being biased by sex [31]. These participants have no motor or cognitive disorders, and differ in age (37.6±13.0 years old), weight (72.9±12.7 kg), height (170.7±6.8 cm), and anthropometric characteristics for the sake of higher data heterogeneity. Prior to the beginning of the experimental session, participants were informed about the execution of the motor tasks.

#### 3.1.2. Walking Actions

The activities to be performed were chosen among the motor disorders that had been treated the most in the IRCCS Maugeri in Bari, Italy. In light of this, in addition to normal walking, four pathological gaits were considered and they are ataxic, equine (foot drop), hemiplegic, and Parkinsonian gaits [32]. The description of these walking patterns is provided in the following.

- *Normal gait* mainly requires alternating flexion–extension movements of the lower limb joints (i.e., hip, knee, and ankle), possibly supported by arm swinging to increase balance.
- *Hemiplegic gait* is the result of poor control of the flexor muscles during the swing phase, as well as spasticity of the extensor muscles involved in the extension of the paretic leg [2]. The knee does not flex normally during swinging, thus causing the leg circumduction [28]. On the paretic side, the hemiplegic patient is prone to raise the shoulder and to keep the arm close to the trunk, flexed, adducted, and with the wrist internally rotated.
- *Equine gait* (foot drop) can be induced by abnormal activity of the plantar muscles in the swing phase, thus resulting in weakness in foot dorsiflexion and an abnormal ankle position for which knee flexion or hyperextension during the stance phase of locomotion are used as compensation [33].
- *Ataxic-cerebellar gait* is characterized by a wide base of support and a low cadence of steps that leads to impaired balance and generates instability [2].
- *Parkinsonian gait* determines the presence of bradykinesia (delayed movement) with short and slow steps, as well as issues in detaching the forefoot [2,34]. Parkinsonian patients even have a forward flexed trunk because of muscle rigidity in this body part and tremors in the hands [31].

#### 3.1.3. IMU Sensors

Wearable IMU sensors (OPAL^TM^, APDM Inc., Portland, OR, USA) were utilized to acquire data from each subject. These devices are automatically calibrated by the Motion Studio system by APDM and are worn by means of elastic bands as Velcro straps to avoid direct contact with the skin and consequent motion artifacts due to friction. Five sensors were selected (see Figure 1) and worn by each participant on both sides of the human pelvis (RP and LP), on the right and left wrists (RW and LW), and on the sternum (S). This distributed placement has been preferred with respect to the tendency by many PGR studies to promote the subject’s comfort in daily life by exploiting the potentiality of e-textiles [35]: more specifically, the sensor on the sternum can be embedded into a smart T-shirt, those on the wrists can be part of a smart bracelet, and those on the pelvis might be inserted in a smart belt. In addition, placing sensors on different body parts is useful to detect a wider myriad of motor actions that involve the upper and lower limbs, whether separately or simultaneously [3,36].

The sensor configuration is almost the same as in a previous work about human activity recognition [3], with the only difference being an additional IMU on the left wrist. Furthermore, this placement enables the detection of pathological behaviors: in fact, the IMUs on the pelvis may reveal the lower limb movements compensating for either hemiplegia or foot drop; on the other hand, the sensor on the sternum allows one to detect Parkinsonian trunk flexion; sensors on the wrists are essential to record the presence/absence of arm movements corresponding to the dominant and/or pathological side (hemiplegia), or the presence of Parkinson tremor. This sensor placement can capture the characteristics of a normal gait too, as already mentioned in a previous work [3].

The experimental data were recorded with a sampling rate of 128 Hz from a 3-axis 14-bit accelerometer to measure linear acceleration, a 3-axis 16-bit gyroscope to acquire angular velocity, and a 3-axis 16-bit magnetometer for magnetic field intensity [37].

#### 3.1.4. Experimental Protocol

The protocol took place at the Laboratory of Movement Analysis of IRCCS Maugeri in Bari, Italy; it encompassed the execution of multiple repetitions of both normal locomotion and the simulation of pathological gaits, meaning that each subject was asked to perform four repetitions for each task. The mimicking quality was qualitatively ensured by a prior familiarization stage, whose duration was not excessively high since physiatrists and physiotherapists executed the tasks. In addition, verisimilitude was further allowed by an expert in the realm of neurorehabilitation, who gave instructions in a video that was taken as a further reference together with the Standford Medicine guidelines [38]. A total of 28 repetitions were recorded for each subject, since hemiplegic and equine gaits were mimicked on each side of the human body to increase the model’s generalizability in recognizing the pathology. In fact, in these cases the asymmetry of motor patterns can be relevant not only for activity classification, but also for effective rehabilitation [9].

Each repetition began with a standing phase of 15 s, proceeded with overground walking on a linear traced path of 7 m, and ended with another standing stage of 5 s, as shown in Figure 1. Note that this path was followed in different directions for the sake of greater data variability. Each subject was told to wait a reasonable time between contiguous repetitions to prevent fatigue [3]. In view of this, the protocol required a total of almost 45 min for each individual.

### 3.2. Classification Pipeline

The pipeline for classifying walking patterns is made up of two parts, which are a preliminary set of preprocessing operations and the implementation of different models for PGR.

#### 3.2.1. Preprocessing

Data from the inertial sensors were initially acquired for each subject and repetition as a matrix of size $N_{s}\times \left(5\cdot 9\right)$, where $N_{s}$ is the number of samples in a single repetition, 5 is the total number of IMU sensors, and 9 is the total number of IMU components (3 for the accelerometer, 3 for the gyroscope, and 3 for the magnetometer). Afterwards, they were processed first with segmentation by visual inspection to exclude both the static period (initial and final standing phases) and the transitional movements (stand-to-walk, walking, and walk-to-stand), isolating the walking patterns of interest.

Accelerometer, gyroscope, and magnetometer values were then normalized for each walking pattern in the range [−1,1] to obtain a uniform representation of the data [3]. Subsequently, a windowing procedure was applied to enlarge the dataset dimensionality by dividing the signal into windows of 128 samples (1 s) with 50% overlap (0.5 s); this window width was chosen so as to capture enough motor patterns without excessively increasing the computational cost [39].

IMU data were resampled both to achieve a uniform sample size and to exploit the whole informative content of the signal [3]. Additionally, the authors examined different combinations of sensors to verify the influence of sensor configurations on the classification performance [3].

#### 3.2.2. Classification Models

In this work, the pipeline for classifying normal and abnormal walking patterns includes three DL-based models:

1. mCNN-1D: a multi-branch one-dimensional convolutional neural network that was already exploited for continuous HAR in a previous work [3];
2. smCNN-1D: a simplified multi-branch one-dimensional convolutional neural network;
3. sCNN-1D: a sequential one-dimensional convolutional neural network.

The authors chose CNN-based architectures in view of their successful application in related works about PGR [22,23,24], together with their efficacy in HAR [3,40].

**Figure 2:**
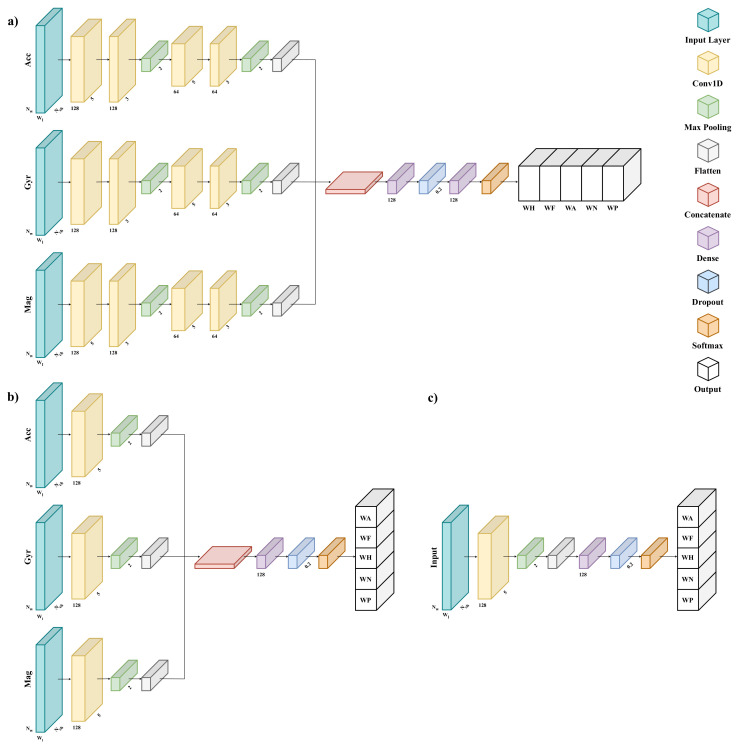
The classification models addressing PGR: (**a**) a multi-branch one-dimensional convolutional neural network; (**b**) a simplified multi-branch one-dimensional convolutional neural network; (**c**) a sequential one-dimensional convolutional neural network. Note that $N_{w}$ is the number of windows in the input dataset, which differs with both subjects and trials; $W_{l}$ is the window length, which is fixed; and $N_{ch}$ is the number of sensor channels.

The mCNN-1D model (Figure 2a) is characterized by the same architecture and hyper parameters as the one used in the previous work [3], with the only difference that the early-stop criterion herein monitors the recall on the validation set to avoid overfitting.

The smCNN-1D model (Figure 2b) differs from mCNN-1D model from the architectural point of view, since it adopts only one 1D convolutional layer, which uses 128 filters with a kernel size of 5, and one fully connected layer with 128 neurons; moreover, the hyper parameters are the same, except for the batch size of 256.

The sCNN-1D architecture (Figure 2c) is inspired by that of the smCNN-1D model. Since it is a sequential CNN, it is not made up of parallel branches, but only of a unique set of layers. Therefore, input data are not split into the different IMU channels (i.e., accelerometer, gyroscope, and magnetometer); on the other hand, such IMU components can be given as input to the model either combined or separately.

For all architectures, to aggregate the data from multiple sensors into one single input, the input layer is fed by a multidimensional array whose shape is $\left(N_{w},W_{l},N_{ch}\right)$, where $N_{w}$ is the number of windows in the input dataset, which may change across subjects and trials; $W_{l}$ is the window length, which is fixed; and $N_{ch}$ is the number of sensor channels, which is equal to the quantity $N_{c}\cdot N_{s}$, given $N_{s}$ the number of sensors in the combination to be evaluated and $N_{c}$ the number of IMU components to be examined. Note that $N_{c}$ can be one for the sCNN-1D when only a single IMU component is used as input to the model; otherwise, it is three.

The classification was designed to recognize five walking patterns, since both hemiplegic and equine walks have not been distinguished based on the affected side.

The data splitting method is the same across all models: the dataset is randomly split across subjects [41,42] such that the data from 60% of them (11 subjects) are assigned to the training set, the data from 20% of them (4 different subjects) to the validation set, and the data from the other 20% (a different 4 subjects) to the testing set. This splitting strategy has already been employed in other studies that are oriented to fall detection [43] and human activity recognition [44], as well as conceived for diagnostic purposes in either the medical field [45,46] or the energetic context [47]. To ensure a fair and unbiased evaluation of each model, the above-mentioned partition is applied ten times and the dataset is split ten times, in each of which different subjects are randomly extracted to constitute the three sets [3,48].

All investigations of the models were conducted on the Google Colab-Pro framework such that each model was trained on a Tesla T4. All the architectures were trained and made inferences by means of Tensorflow, Sklearn, Pandas, and Numpy libraries.

### 3.3. Metrics and Statistics

**Figure 3:**
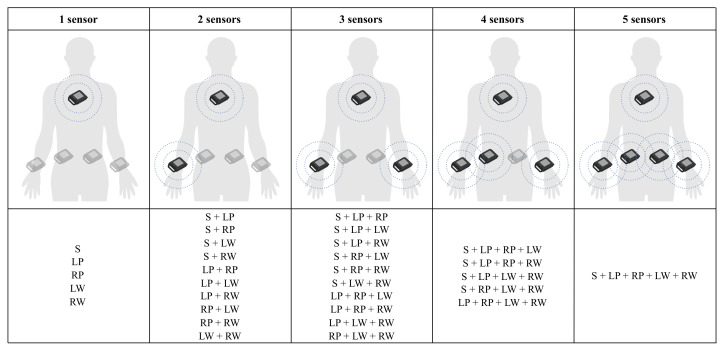
Sensor combinations grouped by the number of sensors, which can be placed at the sternum (S), the left pelvis (LP), the right pelvis (RP), the left wrist (LW), and the right wrist (RW).

Different metrics were used to evaluate the model performance for each sensor combination reported in Figure 3. At first, the test dataset was evaluated in terms of accuracy and recall, whose formulas are reported as follows.

(1) $$\mathrm{Accuracy}=\frac{TP+TN}{TP+TN+FP+FN}$$

(2) $$\mathrm{Recall}=\frac{TP}{TP+FN}$$

where TP, TN, FP, and FN represent true positives, true negatives, false positives, and false negatives, respectively.

On the other hand, the feasibility of the framework in real-time clinical application was investigated by computing the inference time, which is the instant when the data are given as input to the model in which the result classification label is obtained [49].

The normality distribution of these indexes was checked by means of a Shapiro–Wilk test with a significance level of p=0.05. Consequently, for pair-wise comparisons, their values were statistically compared through a non-parametric Wilcoxon signed-rank test for non-normally distributed sets and with a paired t-test (p<0.05) for normally distributed ones.

These analyses were conducted using the Matlab 2022b platform.

## 4. Results and Discussion

In this work, the authors present a framework based on Deep Learning, aiming to perform pathological gait recognition (PGR)—i.e., recognizing healthy and simulated pathological gaits. For this purpose, various architectures based on convolutional neural networks (CNNs) have been implemented, and they are a multi-branch one-dimensional CNN (mCNN-1D), a simplified multi-branch one-dimensional CNN (smCNN-1D), and a sequential one-dimensional CNN (sCNN-1D). Such models have been trained and tested with the data coming from inertial measurement units (IMUs) placed at five body parts, which are the left pelvis (LP), the right pelvis (RP), the left wrist (LW), the right wrist (RW), and the sternum (S). The experimental protocol followed by the subjects entails both the execution of normal gait (WN) and the simulation of four abnormal walking patterns, which are hemiplegic (WH), equine (WF), ataxic (WA), and Parkinsonian (WP) gaits. Three indexes have been employed to evaluate the performance of the proposed workflow: accuracy and recall aim to ascertain the capability to perform PGR, whilst inference time serves to assess its feasibility in a real-time clinical scenario. Note that the classification speed is needed to ensure prompt assistance to any patients; for instance, when they are assisted by a robotic device, the supporting torque provided by the controller should be properly tailored to their disorder [50,51,52,53,54,55,56], which is the one recognized by the classifier. Furthermore, since the self-detection of gait patterns may be difficult, a real-time classification allows for correcting abnormal behaviors [34] or adapting to the patient improvements [36] that would occur during a rehabilitation program. The model is tested for different sensor combinations to establish the optimal configuration in light of the given metrics.

**Figure 4:**
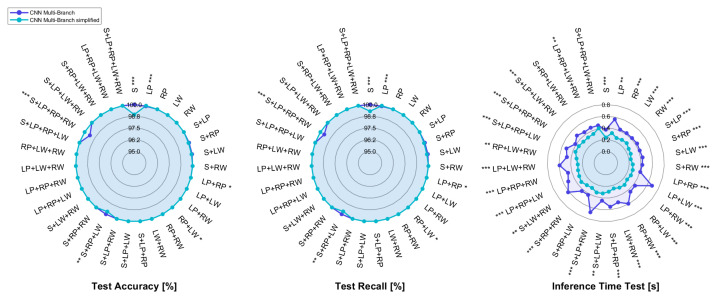
Radar plot comparing metrics computed on the test set for the multi-branch CNN and its simplified version, with *, ** and *** representing statistically significant comparisons with p<0.05, p<0.01, and p<0.001, respectively.

The radar plots reported in Figure 4 compare the results of the mCNN-1D and smCNN-1D architectures, each of which is fed with all three components of the IMU sensors (accelerometer, gyroscope, and magnetometer).

The median of accuracy and recall for the two models is 100% in almost all sensor configurations, despite the dataset imbalance. This could be due to the fact that applying segmentation before normalization allows for the exclusion of all possible motion artifacts that would worsen the classification performance. However, as for the mCNN-1D model, such satisfying results in terms of accuracy are not accompanied by an equally acceptable inference time, since its value approaches 600 ± 100 ms for some combinations, which can be excessive for a real-time application, since it is close to 30% of the execution time of overground walking by impaired subjects [57].

Consequently, the model architecture has been simplified, thus reaching with the smCNN-1D model a test inference time that is significantly lower than the one of the mCNN-1D model in almost all combinations; in addition, the maximum time decreases from approximately 700 ms to about 400 ms for the LP+LW and S+LP+RW sensor pairs. This computational time is even lower than 300 ms for some combinations with either a single IMU or sensor pairs for the simplified multi-branch CNN.

**Figure 5:**
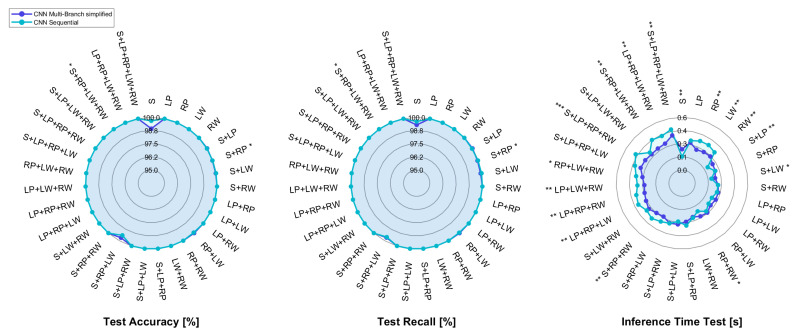
Radar plot comparing metrics computed on the test set for the simplified multi-branch and the sequential CNNs, with *, **, and *** representing statistically significant comparisons with p<0.05, p<0.01, and p<0.001, respectively.

In light of this, the simplified architecture was adopted for the sequential CNN (Figure 2c) as well. The performance achieved with the smCNN-1D and sCNN-1D models, whose input data are the three channels of the IMU sensors, are pictorially depicted in Figure 5.

**Figure 6:**
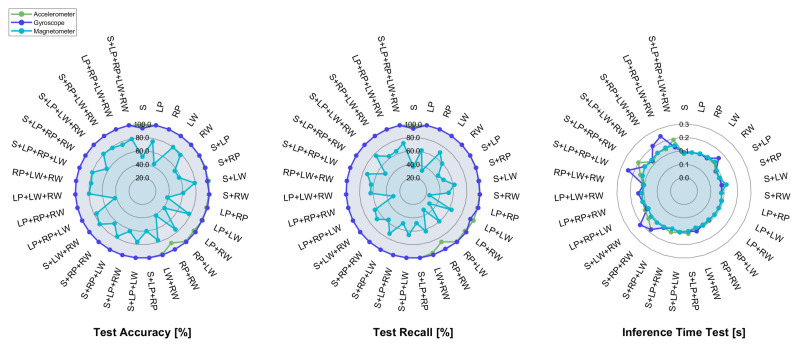
Radar plot of the accuracy, recall, and inference time computed on the test set by feeding the sequential CNN with each IMU component separately.

For parity of accuracy and recall, the inference time does not seem to improve when passing from the smCNN-1D to the sCNN-1D model, except for the sensor on the sternum, in which case the test inference time is about 100 ms. This is presumably due to the use of unnecessary input data; therefore, the sCNN-1D model has been tested for all sensor combinations by separately giving as input each IMU component to the model, with the aim of evaluating which channel (i.e., accelerometer, gyroscope, and magnetometer) leads to the best model performance. The consequent results in terms of accuracy, recall, and inference time are pictorially shown with the radar plots reported in Figure 6.

The outcomes show better performance in both accuracy and recall for the accelerometer and gyroscope, whereas the outcome worsens when passing the magnetometer entry as input to the model, since the recall is about 80% only in three combinations (i.e., S+LW+RW, S+LP+LW+RW, S+RP+LW+RW). Moreover, the lowest values of accuracy are achieved for the combinations with one sensor placed at either the sternum or the right pelvis; instead, the accuracy for the LW+RW pair is higher than the one related to the LP+RP pair. Hence, it can be argued that the information carried by the magnetometer placed on the wrist raises the performance with respect to the magnetometers of the IMU located at the sternum and pelvis, which are not equally discriminative. In fact, the motor behavior of human wrists is different for each type of walking action: it is stationary on the affected side in the hemiplegic gait close to the sternum, and it follows hand tremors in the Parkinsonian gait and arm sway in normal walking.

The outcome of the above-mentioned model in terms of inference time lies in the range of [100,200] ms for almost all the three components, except for some combinations in which it slightly exceeds 200 ms in the case of the accelerometer and gyroscope. This outcome is more compliant to real-time requirements, since it at most equals 10% of the execution time of pathological walking [57]. Therefore, the sequential CNN model using either the accelerometer or the gyroscope seems to provide the best compromise among all the above-mentioned metrics.

**Figure 7:**
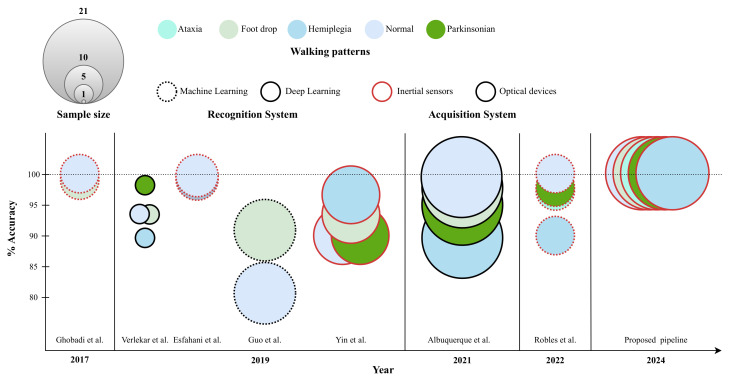
Related works simulating abnormal walking patterns [2,6,7,11,22,23,24].

Figure 7 and Table 1 report the average accuracy in distinguishing normal and simulated pathological gaits to compare the proposed framework with similar studies in the literature: all walking patterns have been classified with an average accuracy of 100%, thus outperforming related works.

For instance, Robles et al. [2] utilized Artificial Neural Networks (ANNs) to classify simulated gait disorders (Parkinsonian, ataxic, and hemiplegic gaits) by analyzing accelerations of the center of mass of ten healthy subjects. An average accuracy of 99.8%, 90.3%, 97.3%, and 98.1% was obtained for the classification of normal, hemiplegic, ataxic, and Parkinsonian gaits, respectively. Ghobadi et al. [7] discriminated normal gait from foot drop (FD) patterns, which were mimicked by ten healthy subjects, with an average accuracy of 99.6% and 98.7% (respectively) by training an SVM with Mahalanobis distance-based features extracted from the IMU signals of the subject’s right leg. Esfahani et al. [11] employed various ML-based algorithms (e.g., ANNs, SVM, and kNN), which were fed with the data of eleven healthy subjects coming from a smart textile system, thus being able to differentiate between normal and hemiplegic gaits with an average accuracy of 99.6% and 99.4%, respectively.

However, such studies performed the actual classification after a time-consuming feature engineering procedure. On the other hand, other related works relied upon DL architectures to automatically extract features instead of handcrafting them [58]. For example, Guo et al. [6] exploited not only SVM, but also Bidirectional Long Short-Term Memory (BiLSTM) networks to classify normal and mimicked foot drop walking patterns based on the images acquired with a single RGB-D camera; in so doing, average accuracies of 81.24% and 92.9% were, respectively, achieved for the normal and the pathological gaits, which were simulated by sixteen healthy subjects. Albuquerque et al. [24] recruited twenty-one healthy subjects and developed a remote pathological gait classification system for discriminating normal, hemiplegic, foot drop, and Parkinsonian gaits, thus achieving average accuracies of 99%, 89%, 97%, and 95%, respectively. The performance of these two works is almost comparable with our study except for the recognition of one class (i.e., normal and hemiplegic gait, respectively), which is mainly confused with similar walking patterns that are not fit within the focus of our study. Verlekar et al. [22] trained CNN-based models with gait energy images, which were determined from a dataset of five healthy subjects; in so doing, it was possible to distinguish normal from hemiplegic, foot drop, and Parkinsonian gait sequences with an average accuracy of 94%, 87%, 94%, and 98%, respectively. In spite of these outcomes, in their mind the extracted features do not perfectly suit the field of gait classification, which may explain misclassifications between similar walking patterns (e.g., foot drop and hemiplegia).

Moreover, these latter studies addressed PGR with data coming from optical trackers, which may be limited in real-world scenarios. To the best of our knowledge, only one work has employed IMU data to recognize simulated pathological gaits by means of DL architectures. Yin et al. [23] utilized two accelerometers mounted on the lower limbs to classify normal and simulated pathological gaits through both ML (i.e., ANN) and DL models (LSTM and CNN); in so doing, normal, hemiplegic, and Parkinsonian motor patterns were discriminated with average accuracies of 89%, 91.3%, and 78.6%, respectively. Despite the promising performances, they are yet lessened by the proposed workflow, arguably due to the order of preprocessing operations (i.e., normalizing signals before segmenting), which may introduce motion artifacts worsening classification performance; in addition, that investigation was conducted on a lower sample size (i.e., number of participants) with the same sensor type (i.e., inertial measurement units) and did not consider the same walking patterns as the authors’ work.

## 5. Conclusions

This work presents a Deep Learning-based framework aimed at the classification of normal and abnormal walking patterns by means of multiple convolutional neural networks, which are fed with the data coming from inertial sensors worn at different locations of the human body.

Given the promising performance of the models used in terms of accuracy and inference time, the authors claim the effectiveness of the proposed workflow in discriminating motor patterns. The instructions of an expert clinician for a realistic gait disorder simulation contributed to the quality of classification results. However, the framework has been tested only on data related to locomotor actions, both normal and abnormal, performed by healthy subjects, since this work was conceived as a feasibility study before application on actual pathological gaits. Therefore, the proposed workflow should be evaluated by studying data coming from people actually affected by gait disorders to test its usefulness in a clinical scenario. In so doing, even the severity rating of such neuromotor disorders as Parkinson’s disease [12,13,14,15,16] and ataxia [17] can be explored. Furthermore, since the motor actions have been performed by people with advanced clinical knowledge, a successive experimental campaign could include subjects with no expertise in gait disorders for the sake of even higher data variability [59].

Secondly, only five walking patterns have been considered, since the samples of the hemiplegic and equine gaits have not been distinguished according to the emulated affected side; hence, a seven-class classification may be pursued to investigate whether the framework effectively performs PGR even in cases of unilateral gait disorders, which may be useful for the corresponding rehabilitation [9].

The type of abnormal walking patterns considered in this work, though in line with most of the related works, is restricted to evident motor behaviors. Therefore, for the sake of even greater usefulness in a clinical scenario, less obvious motor patterns such as walking tremors [60] should be considered in the future. Another example is given by Parkinsonian tremors, which could be further addressed in view of its previous successful simulation by healthy subjects with slight amplitude differences with respect to actual pathological subjects [61]. This may be carried out by conceiving some indexes to measure the deviation of an individual’s gait pattern from the range typical of a healthy subject, thus tracking the patients’ rehabilitative progress and the effectiveness of therapies [28,31,62,63].

Future research could also aim to develop an integrated explainable framework that combines the strengths of attention mechanisms [41,64] and Layer-wise Relevance Propagation [65]. These methods help in understanding which parts of the input data (i.e., IMU component) contribute the most to the model predictions.

With this in mind, the proposed gait recognition system can be enhanced to pursue its seamless integration into either the clinical realm or a home application [36]. This may be achieved by embedding the inertial sensors within e-textiles to increase the subject’s comfort either in a rehabilitative path or in a remote-monitoring application.

## Figures and Tables

**Table 1:** Relevant features from related works, reported for comparisons among workflows.

	Ghobadi et al. [7]	Verlekar et al. [22]	Eshefani et al. [11]	Guo et al. [6]	Yin et al. [23]	Albuquerque et al. [24]	Robles et al. [2]	Proposed Framework
Subjects	10	5	11	16	15	21	10	19
Input data	IMU signals	Data from images	Data from smart textiles	Data from images	IMU signals	Data from images	Data from accelerometer	IMU signals
Classifier type	ML	DL	ML	ML DL	DL	DL	ML	DL
Classifier	SVM	CNN	kNN, LDA, SVM, ANN	SVM BiLSTM	ANN, LSTM, CNN	CNN	ANN	CNN
Normal	99.6%	94.0%	99.6%	81.24%	89.0%	99.0%	99.8%	100%
Hemiplegia	ND	87%	99.4%	ND	91.3%	89.0%	90.3%	100%
Ataxia	ND	ND	ND	ND	ND	ND	97.0%	100%
Foot drop	98.7%	94.0%	ND	92.9%	ND	97.0%	ND	100%
Parkinsonian	ND	98.0%	ND	ND	78.6%	95.0%	98.1%	100%

## Data Availability

The data presented in this study are available on request from the corresponding author. Moreover, some reference videos of the simulated gait disorders can be provided upon request.
